# Suprasellar cysts: clinical presentation, surgical indications, and optimal surgical treatment

**DOI:** 10.1186/1471-2377-11-52

**Published:** 2011-05-18

**Authors:** Song-Bai Gui, Xin-Sheng Wang, Xu-Yi Zong, Ya-Zhuo Zhang, Chu-Zhong Li

**Affiliations:** 1 Department of Neurosurgery, Beijing Tiantan Hospital, Capital Medical University, Beijing, China; 2 Beijing Neurosurgical Institute, Beijing, China

**Keywords:** Suprasellar cyst, clinical presentation, endoscopic fenestration, outcome

## Abstract

**Background:**

To describe the clinical presentation of suprasellar cysts (SSCs) and surgical indications, and compare the treatment methods of endoscopic ventriculocystostomy (VC) and ventriculocystocisternotomy (VCC).

**Methods:**

We retrospectively reviewed the records of 73 consecutive patients with SSC who were treated between June 2002 and September 2009. Twenty-two patients were treated with VC and 51 with VCC. Outcome was assessed by clinical examination and magnetic resonance imaging.

**Results:**

The patients were divided into five groups based on age at presentation: age less than 1 year (n = 6), 1-5 years (n = 36), 6-10 years (n = 15), 11-20 years (n = 11), and 21-53 years (n = 5). The main clinical presentations were macrocrania (100%), motor deficits (50%), and gaze disturbance (33.3%) in the age less than 1 year group; macrocrania (75%), motor deficits (63.9%), and gaze disturbance (27.8%) in the 1-5 years group; macrocrania (46.7%), symptoms of raised intracranial pressure (ICP) (40.0%), endocrine dysfunction (40%), and seizures (33.3%) in the 6-10 years group; symptoms of raised ICP (54.5%), endocrine dysfunction (54.5%), and reduced visual field or acuity (36.4%) in the 11-20 years group; and symptoms of raised ICP (80.0%) and reduced visual field or acuity (40.0%) in the 21-53 years group. The overall success rate of endoscopic fenestration was 90.4%. A Kaplan-Meier curve for long-term efficacy of the two treatment modalities showed better results for VCC than for VC (p = 0.008).

**Conclusions:**

Different age groups with SSCs have different main clinical presentations. VCC appears to be more efficacious than VC.

## Background

Suprasellar cysts (SSCs) are benign developmental collections of cerebrospinal fluid (CSF). Their presence in utero and their high prevalence in children who have no history of trauma support the assumption that they are congenital [1-3]. Although their true histological nature is rarely known because their membranes are not often analyzed histologically, it is reasonable to think that the majority are arachnoid cysts. Suprasellar cysts constitute approximately 9% of all arachnoid cysts [4]. These cysts progressively enlarge from an abnormality in the membrane of Liliequist or in the interpeduncular cistern [5,6]. Typically, SSCs expand from the prepontine space, displacing the floor of the third ventricle upwards, the pituitary stalk and optic chiasm upwards and forwards, and the mammillary bodies upwards and backwards. As the cyst increases in size it fills and occludes the third ventricle, and distorts and blocks the aqueduct, which finally results in hydrocephalus.

A suprasellar cyst can be a communicating cyst with a valve at the penetration of the basilar artery (BA) through the prepontine arachnoid membrane or be a noncommunicating cyst [7]. Miyajima et al proposed a new classification of suprasellar arachnoid cysts: 1) a noncommunicating intra-arachnoid cyst of the diencephalic membrane of Liliequist and 2) a communicating cyst that is a cystic dilation of the interpeduncular cistern [6]. Intra-arachnoid cysts are noncommunicating, the basilar bifurcation pushes posteriorly against the brainstem. Cystic dilation of the interpeduncular cistern occurs between the two leaves of Liliequist's membrane; the basilar bifurcation lies inside the cyst.

Endoscopic surgery has been advocated for treatment of arachnoid cysts. Shim et al studied 209 patients with arachnoid cysts and suggested that endoscopic procedures were superior to large craniotomy or shunting, which are associated with a number of complications [8]. For patients with a SSC who have hydrocephalus, endoscopic surgery is the primary treatment. However, for patients with a SSC who do not have hydrocephalus the choice of treatment is less clear. Because SSCs are rare this has prevented studies with large numbers of patients. Most series have included only a few patients. For example, Crimmins et al reported on 7 patients treated with ventriculocystostomy (VC) and 13 patients with ventriculocystocisternotomy (VCC) [9]. They found VCC had a higher success rate but the difference was not statistically significant. Therefore, because of the lack of data some facets have not been thoroughly characterized. Certain issues remain controversial, including surgical indications and the type of endoscopic surgery (VC or VCC).

In this report, we describe a series of 73 patients with SSCs who were treated at our hospital. The aims of this study were to determine the peak age of patients with SSCs at diagnosis, describe the main symptoms of different age groups, identify which symptoms can be improved after surgical treatment and which never regress, determine the surgical indications, and compare VC with VCC to assess which endoscopic treatment method is better.

## Methods

### Patient population

This retrospective study was approved by the Institutional Review Board of Beijing Tiantan Hospital. The study included 73 consecutive patients with SSCs who underwent endoscopic VC (n = 22) or VCC (n = 51) between June 2002 and September 2009. The patients were selected for VC or VCC on the basis of the experience of the neurosurgeons. During the early period the majority of patients underwent VC but as the neurosurgeons gained experience over time there was an increase in the proportion of patients undergoing VCC which is theoretically considered to be the more reliable procedure. Information obtained from the patients' records included age at presentation, symptoms and signs, treatment modalities, postoperative complications, and postoperative clinical and magnetic resonance imaging (MRI) evaluations. Patients were excluded if they had a lateral ventricle that was not large enough for endoscopic surgery, had no symptoms, or had previously undergone shunting.

All of the patients were treated for the first time and they all had hydrocephalus. Before and after surgery the patients underwent MRI. Standard T1-weighted axial, coronal, and sagittal sequences were assessed to establish the diagnosis of SSC. Because an arachnoid cyst most closely approximates an ellipsoid shape, we calculated the cyst volume based on the following formula: volume = (4π/3)(r1)(r2)(r3), with each radius (r) being determined from the maximal diameter in the anterolateral, lateral, and vertical planes. The size of the lateral ventricle was estimated using the frontal and occipital horn width ratio (FOHWR) [10].

### Endoscopic Technique

The endoscope was introduced through a point about 3 cm lateral to the midline and about 1 cm anterior to the coronal suture. As determined on preoperative sagittal MRI, the position of the point varied according to the anatomy of the cyst and ventricle so that we could obtain an optimal trajectory to make the aqueduct visible through the foramen of Monro and the endoscope could be inserted along a trajectory that would enable fenestration of both the apical and basal cyst membranes with minimal anterior-posterior manipulation.

**Figure 1:**
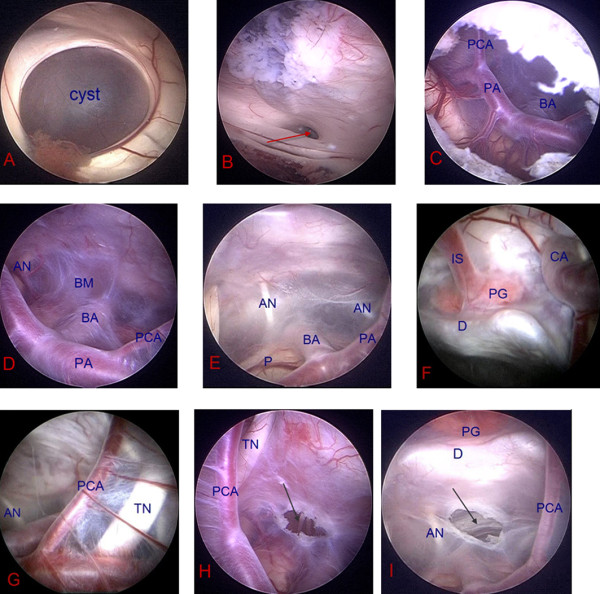
**Intraoperative photographs of the SSC**. A: Bluish-colored apical dome of the suprasellar cyst. B: The capsule of the cyst was shrunk down until the aqueduct was clearly visible. C: A large fenestration was made in the apical membrane of suprasellar cyst. D, E, F, G: The displaced cisternal contents in the cyst. H, I: Fenestration of the basal cyst membrane. BA = basilar artery; PA = P1 segment of posterior cerebral artery; PCA = posterior communicating artery; AN = abducent nerve; BM = basal membrane of the arachnoid cyst; P = pons; IS = infundibular stalk; PG = pituitary gland; CA = carotid artery; D = dorsum sellae; TN = trigeminal nerve; Aqueduct of mesencephalon (red arrow); Fenestration of the basal membrane of the cyst (black arrows).

Generally, the endoscope was inserted along a line directed to the imaging line which connected the bilateral external auditory foramens, but the direction deflected slightly to the median line to prevent injury to the thalamus. After using standard anatomical landmarks to confirm visual entry into the right lateral ventricle, the endoscope was advanced to the foramen of Monro, allowing identification of the bluish-colored apical dome of the arachnoid cyst (Figure 1A). First, small blood vessels on the cyst wall were obliterated and a fenestration in its apical membrane was made using scissors. Second, the capsule of the cyst was shrunk down with the aid of an endoscopic bipolar coagulator until the aqueduct was clearly visible (Figure 1B), which can prevent the persistent redundant wall of the cyst from free-floating in the third ventricle. Third, the cyst wall was resected as much as possible to make a large fenestration in its apical membrane using scissors (Figure 1C). This completed the VC procedure.

To perform VCC, the endoscope was advanced into the cyst, allowing visual inspection of the displaced cisternal contents (Figure 1D,E,F,G). The endoscope was further advanced to the basal cyst membrane, where a cystocisternotomy was performed anterior to the BA (Figure 1H,I). At this point multiple fenestrations should be made in avascular portions of the membrane by using blunt biopsy forceps and scissors between cranial nerves exiting the brainstem. The fenestrations should be as large as possible and as many as possible. In some cases, the lower portion of the cyst formed a relatively flat membrane between the clivus and the pons, and a second fenestration was easy and safe to perform. In the cases where the inferior wall of the cyst extended for some considerable distance and was plastered to the clivus before being reflected onto the basilar artery and the pons, a cystocisternotomy can be performed by making a fenestration against the clivus in the lower portion of the cyst which is attached to the clivus.

### Follow-up evaluation

Postoperatively, patients underwent clinical and MRI evaluations. Clinical outcome was recorded as improved, unchanged, or deteriorated. Clinical improvement was defined as partial or complete relief of symptoms; deterioration was defined as a progression of clinical symptoms.

Computed tomography (CT) or MRI of the head was performed during the first week postoperatively before discharge. All patients underwent MRI during the follow-up period at 3 and 12 months, and then annually to compare the pre- and post-fenestration cyst size, ventricle size, and mass effect. Cine-MRI was used in some patients to look for cerbrospinal fluid (CSF) flow artifact at the fenestration sites as a demonstration of patency.

**Figure 2:**
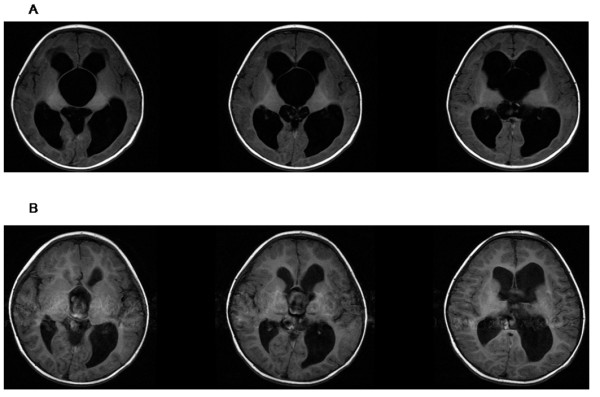
**Axial magnetic resonance images obtained in a patient who harbored SSC and hydrocephalus**. A: Preoperative axial T1-weighted image (male, 1 year old). B: Postoperative axial T1-weighted image (25 months later).

**Figure 3:**
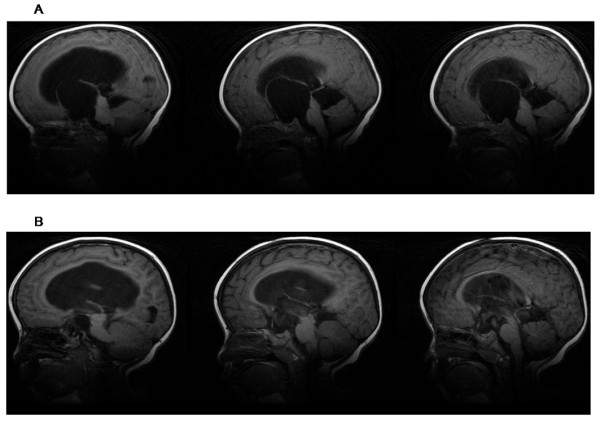
**Sagittal magnetic resonance images obtained in same patient**. A: Preoperative sagittal T1-weighted image (male, 1 year old) B: Postoperative sagittal T1-weighted image (25 months later).

A successful operation was defined as improvement in symptoms and demonstration of the following features on postoperative MRI (these features can accurately indicate the effectiveness of the endoscopic procedures): (1) reductions in size of the cysts (Figure 2A,B); (2) improvement in chiasmatic and mammillary body distortion and pontine effacement (Figure 3A,B). Changes in size of the lateral ventricle were used to assess outcome as a supplementary measure because such changes are not very clinically significant.

### Statistical analysis

Data were presented as mean ± standard deviation or frequencies. The success rates of VC and VCC were compared using a Kaplan-Meier curve for long-term efficacy. The log-rank test was used to compare long-term efficacy of the two treatment modalities. Data were analyzed using SPSS 15.0 software (SPSS, Inc., Chicago, IL, USA). A p-value < 0.05 was considered statistically significant.

## Results

### Preoperative evaluation

Among the 73 patients, 39 were male and 34 female. Age at diagnosis ranged from 6 months to 53 years. The patients were divided into five groups based on age at presentation (Table 1): age less than 1 year (n = 6, 8.2%),1-5 years (n = 36, 49.3%),6-10 years (n = 15, 20.5%),11-20 years (n = 11, 15.1%),21-53 years (n = 5, 6.8%). The clinical symptoms and signs at presentation of each age group are listed in Table 1.

**Table 1:** Symptoms and clinical signs of different age groups

	Age less than 1 year(n = 6)	1-5 years (n = 36)	6-10 years (n = 15)*	11-20 years (n = 11)†	21-53 years (n = 5)
Macrocrania	6	27	7	2	--

Motor deficits	3	23	4	3	1

Microplasia		--	3	4	--

Headache/vomiting/drowsiness		5	6	6	4

Head bobbing		3	2	1	--

Gaze disturbance	2	10	3	--	--

Reduced visual field or acuity		5	3	4	2

Seizures		4	5	1	--

Precocious puberty		2	4	3	--

Delayed puberty(primary amenorrhea)		--	--	2	--

* One patient had both microplasia and precocious puberty

† Two patients with delayed puberty and one patient with precocious puberty also had microplasia

The MRI features found consistently in all patients included (1) cystic masses with an MRI signal intensity similar to CSF on all sequences; (2) marked dilatation of the third and lateral ventricles producing the typical Mickey Mouse appearance on axial imaging; (3) on sagittal T1-weighted MR images, the suprasellar cysts displayed the following three diagnostic MRI features: vertical displacement of the optic chiasm/tracts; upward deflection of the rostral mesencephalon and mammillary bodies; and effacement of the ventral pons.

### Postoperative Evaluation

Of the 22 VCs, 18 were effective and 4 failed. In two of the unsuccessful procedures, treatment failure was immediately apparent postoperatively. Repeated VCC was performed after the failure of previous endoscopic treatments in one patient and the symptoms improved after the procedure. For the other patient, who had the preoperative symptom of isolated precocious puberty, symptoms did not improve despite a decrease in cyst volume, and no further surgery was performed. The other two patients had clinical improvement after surgery, but cyst regrowth and symptoms recurred at 8 months and 1 year, respectively. Both underwent VCC and the symptoms improved in one patient whereas in other patient the second endoscopic procedure failed and the symptoms improved after ventriculoperitoneal (VP) shunting was performed.

Of the 51 VCCs, 48 were successful and 3 failed. One patient who had an unsuccessful procedure, had the symptom of isolated microplasia, and although the cyst volume decreased, the symptom did not improve. This patient did not undergo further surgery. Among the other two patients with unsuccessful procedures, the treatment failure was immediately apparent postoperatively in one and in the other failure was not recognized until after 15 months of apparently good function. The symptoms improved after VP shunting in both patients.

Among the 69 patients whose first endoscopic procedure was successful, 21 had symptoms of elevated ICP (headaches, dizziness, vomiting and drowsiness) and 20 (95.2%) of these patients had improvement of these symptoms. The improvement rate of motor deficits (ataxic gait, astasia, tremor of the limbs, etc) was 91.2% (31/34). Head bobbing improved in all six (100%) affected patients. Thirteen (92.9%) of 14 of patients who had symptoms of reduced visual field or acuity had complete disappearance or marked reduction of these symptoms. Also, the growth in head circumference stopped after fenestration in all patients. Ten patients had epilepsy before surgery; seizures in eight of them were reduced or disappeared while they were taking oral anti-epileptic drugs. Preoperative endocrine disorders (precocious puberty, microplasia and delayed puberty) and gaze disturbance (ocular bobbing and heterotropia) did not resolve postoperatively in any patient. Furthermore, five patients who did not have endocrine dysfunction before surgery developed endocrine dysfunction during postoperative follow-up (one patient had microplasia, three had precocious puberty, and one had both) even though they had successful surgery.

In the 69 patients whose first endoscopic procedure was successful, postoperative MRI demonstrated several features that are indicative of effective therapy: (1) The cysts shrank in all 69 patients; (2) The optic apparatus and mammillary bodies reoriented to a more horizontal position and pontine deformation improved in all 69 patients (Figure 3A,B); (3) There was neuroimaging evidence of CSF flow through both the apical and basal fenestrations sites on cine mode MRI; (4) The size of the ventricle decreased in 46 patients and stabilized in 23 patients.

**Figure 4:**
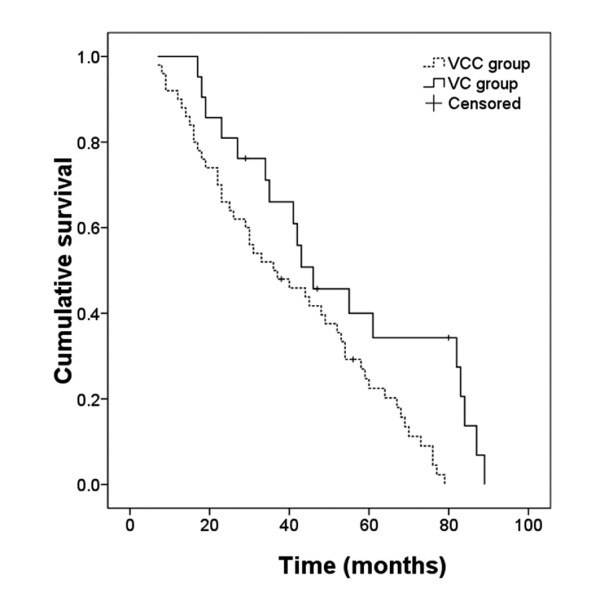
**Kaplan-Meier curve for long-term cumulative survival. **VC = ventriculocystostomy; VCC = ventriculocystocisternotomy.

Excluding the two patients whose preoperative symptoms were isolated endocrine dysfunctions, the VCC group included 50 patients and 48 had a successful operation; the VC group included 21 patients and 18 had a successful operation. The mean follow-up duration was 49.6 ± 25.2 months and 39.4 ± 22.2 months in the VC and VCC groups, respectively. A Kaplan-Meier curve for long-term efficacy of the two treatment modalities showed better results for VCC than for VC (p = 0.008) (Figure 4).

There was no mortality or morbidity in our series. Following VCCs there were 13 complications: four transient episodes of sixth nerve palsy, five subdural fluid collections (with no symptom and no need for any treatment), one episode of polydipsia and polyuria (symptoms disappeared 15 days after the operation), and three seizure disorders (patients took anti-epileptics orally for 3 months and there was no recrudescence after drug discontinuance during the follow-up period). Following VCs there were four complications: one CSF leak requiring a lumbar drain for 4 days, one seizure disorder (patients took anti-epileptics orally for 3 months and there was no recrudescence after drug discontinuation during the follow-up period) and two subdural fluid collections (with no symptom and no need for any treatment).

## Discussion

### Age group and symptoms

We found that in infants, the main clinical presenting symptoms were macrocrania and motor deficits. In children and adolescents, the main presenting symptoms were elevated ICP, endocrine dysfunction, and reduced visual field or acuity. In adults, the main presenting symptom was elevated ICP. These age-related findings should be useful in the clinical setting for diagnosis of SSCs. We also found that a higher proportion of our patients were male (53%). Pierre-Kahn et al also reported a male preponderance in patients with SSCs, but the proportion was higher (61%) [7].

### Methods of diagnosis

Suprasellar cysts can mimic a dilated third ventricle and can be misdiagnosed on CT scans as aqueduct stenosis. Such problems can be avoided with the use of MRI, which is the key to diagnosing SSCs. Wang et al reported that on MRI, SSCs have three characteristic features: vertical displacement of the optic chiasm/tracts, upward displacement of the mammillary bodies, and ventral pontine displacement [11]. These findings differ from those of noncommunicating hydrocephalus caused by aqueduct stenosis in which the mammillary bodies and the third ventricular floor are inferiorly displaced.

### Surgical indications

There is controversy about whether large but asymptomatic SSCs require surgery and which symptoms indicate that surgery is needed. With regard to asymptomatic SSCs, regardless of their size, if they do not evolve they do not need treatment. We found one case of SSC which disappeared spontaneously during follow-up, and other authors have also reported that suprasellar cysts may disappear spontaneously [12,13]. A possible mechanism for SSC resolution is the formation of a communication between the cyst and the ventricle or basal cistern because of a tearing of a weakened cyst wall or a widening of the slit-valve-like arachnoid membrane around the basilar artery may occur prior to the disappearance of the SCC [13]. Most of the cysts found during the prenatal period decreased in size, disappeared completely, or stabilized without associated clinical deficits during follow-up [2,9]. Therefore, a conservative approach should be chosen if a child with a cyst has no symptoms, is neurologically intact, and undergoes close clinical and radiological observation [7,9,14].

In our series, symptoms of elevated ICP, motor deficits, head bobbing, and reduced visual field or acuity all improved in patients who underwent surgery which was successful, but endocrine disorders and gaze disturbance never regressed. The lack of improvement in gaze disturbance is in contrast to the usual finding that sixth nerve palsy usually improves after treatment for hydrocephalus. It may be that when cranial nerve paralysis is caused by intracranial hypertension that improvement is likely after treatment but when the paralysis is related to SSCs the paralysis is often caused by direct compression and therefore the paralysis involves a different mechanism than that involved with intracranial hypertension and the recovery rate of eye movement disorders following surgery is relatively poor. Our finding that gaze disturbance did not regress following treatment is consistent with a report by Erşahin et al that endoscopic treatment of suprasellar arachnoid cysts failed to improve bilateral sixth nerve palsy in one patient [15]. There were five patients in our study who had endocrine dysfunction during postoperative follow-up despite successful surgery. This observation has also been reported by other authors [7,9]. Therefore, with regard to which symptoms indicate surgery, we would argue that surgical indications should include signs of elevated ICP (including increased head circumference), motor deficits, head bobbing, and reduced visual field or acuity. Only six patients in our study had head bobbing. However, three cases have been reported in the literature of patients with bobble-head syndrome who have been successfully treated with endoscopic surgery for suprasellar arachnoid cysts providing additional evidence that head bobbing is an indication for surgery [16,17]. Also, surgery is indicated by progressive enlargement of the cyst even if patients are asymptomatic. Contraindications to surgery include the absence of symptoms and isolated endocrine dysfunction or gaze disturbance. In addition, it is a common observation that behavioral difficulties and mental retardation never regress postoperatively [9]. Most arachnoid cysts that involve the interpeduncular cistern would be classified as supracellar cysts, however, some may be pure interpeduncular cysts [18]. Such pure interpeduncular cysts tend to remain stable over time and therefore a conservative treatment strategy has been recommended [18].

### Choice of surgical procedure

**Figure 5:**
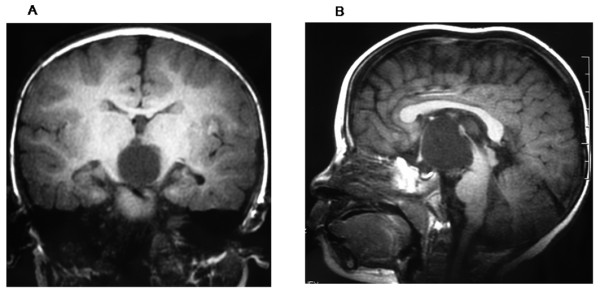
Suprasellar cysts without hydrocephalus.

Suprasellar cysts can be divided into two types: with hydrocephalus or without hydrocephalus (Figure 5A,B). It is rare for a patient with a suprasellar cyst who does not have hydrocephalus to need surgical treatment. The therapeutic goals are to remove the mass effect of these cysts by resection, fenestration, or shunting of the cyst and relieve the hydrocephalus.

Numerous modalities of surgical treatment including microsurgical excision or fenestration, endoscopic fenestration, shunting of the cyst and/or ventricular system, and percutaneous ventricle-cystostomy have been used for the treatment of the SSCs.

For patients without hydrocephalus, an endoscopic procedure cannot be performed, so there are two treatment choices: shunting of the cyst and/or ventricular system and microsurgical excision or fenestration of the cyst. For patients with hydrocephalus, there are three treatment choices: endoscopic fenestration of the cyst, shunting of the cyst and/or ventricular system, and microsurgical excision or fenestration of the cyst.

The direct approach to the cyst for microsurgical excision or fenestration of the cyst has been performed by the subfrontal, pterional, transventricular, and transcallosal routes [19-22]. This treatment is rather invasive with more postoperative complications because of the deep location of these cysts.

The common problems with any shunting procedure, such as shunt malfunction, infection, and shunt dependency are well known. Also, the success rate of the VP shunt as a definitive treatment for SSCs is reported to be only 10% [21].

Recently, advances in endoscopy have brought endoscopic fenestration to the forefront of the management algorithm for SSCs with hydrocephalus and the procedure has been demonstrated to be safe and effective [9,11,15,23,24]. An analysis of 23 reviewed series from 1980 to 2007 showed that a good clinical-radiological outcome was noted in 30 of 38 (79%) patients treated by craniotomy and cyst excision, in 18 of 21 (85.7%) after shunt of the cyst, in 12 of 15 (80%) after percutaneous ventricle-cystostomy, and in 92 of 102 (90%) after endoscopic fenestration [24]. More recently, Yadev et al also reported good results with endoscopic surgery in a series of 12 patients [25]. In our series, the overall success rate was 90.4%. Therefore, based on the evidence, we think that endoscopic surgery should be the first choice in the management of SSCs with hydrocephalus at the present time.

Endoscopy is impractical in the presence of tiny ventricles, so for the patients without hydrocephalus, Crimmins et al believe that with the advances in microsurgical techniques, open marsupialization of the cyst into the basal cisterns is a more effective solution that should be preferred to shunt insertion [9].

Primary endoscopic procedures failed in seven patients in our series. Considering the frequency of shunt complications, we would still prefer VCC to shunt implantation in cases of VC treatment failure. Among the three patients with endoscopic VCC after VC failure in our series, two patients had clinical improvement.

In patients with VCC treatment failure, we considered that the persistence of hydrocephalus in these patients may have been due to impaired CSF resorption. Therefore, for such patients, we prefer shunt implantation to repeated VCC or direct approach to the cyst through craniotomy.

### Choice of endoscopic surgical procedure

There are presently three types of endoscopic surgical procedures: 1)VC, the goal of VC is to establish communication between the cyst cavity and ventricles; 2) VCC, the goal of VCC is to open the cyst into both the ventricles and cisterns; 3) endoscopic fenestration of suprasellar cysts followed by enough shrinkage coagulation of the cysts, this procedure is done after fenestration between the cyst cavity and ventricles is finished [26]. Among the three methods, VCC and VC are most often used, endoscopic fenestration of SSCs followed by enough shrinkage coagulation of the cysts has only been reported by one author [26].

Currently, the main controversy is about whether VCC is preferable to VC alone. Gangemi et al reviewed 176 patients with suprasellar arachnoid cysts treated by different surgical procedures from 23 series reported after 1980 [24]. The endoscopic procedure was VC in 49 patients and VCC in 53. The rate of clinical-radiological improvement was higher after VCC (94.3%) than after VC (85.7%). Crimmins et al also found that the failure rate of VC to be higher than that of VCC [9]. In our series, VCC was also more frequently effective (48 of 50) than VC (18 of 21). We think that there are two reasons to explain this result: First, the superior fenestration tends to close, regardless of whether a single or dual fenestration is performed because stretching of the third ventricle creates excess tissue that can overlap and seal the fenestration after the operation. The persistence of the basal opening, even in the face of secondary closure of the apical fenestration, allows adequate cyst decompression into the basal cisterns, thus decreasing the risk of recurrence [27]. Second, chronic midbrain compression by the SSC may lead to secondary aqueductal occlusion. In this scenario, apical membrane fenestration alone, although allowing for adequate cyst decompression, may not result in extraventricular CSF flow. The basal membrane fenestration serves the purpose of allowing trapped fluid to pass into the basal cisterns and bypass the occluded aqueduct altogether.

For the third method, endoscopic fenestration of SSCs followed by enough shrinkage coagulation of the cysts, we think that it is not reliable enough because the method also cannot solve the problems related to secondary aqueductal occlusion.

A limitation of the this study was its retrospective design. Another limitation was the small number of patients in the youngest and oldest age groups.

## Conclusions

Most patients with SSCs at diagnosis are younger than 5 years. Different age groups have different main clinical presentations. Surgical indications should include signs of increased ICP, motor deficits, head bobbing, reduced visual field or acuity, and progressive enlargement of the cyst even if the patients were asymptomatic. Contraindications to surgery include the absence of symptoms and isolated endocrine dysfunction or gaze disturbance. Endoscopic VCC should be performed as the first surgical procedure in all cases with hydrocephalus. Patients who do not improve after the VCC procedure may be treated by VP shunting.

## Competing interests

The authors declare that they have no competing interests.

## Authors' contributions

SG carried out conception, acquisition of data, design, analysis and interpretation of data, drafting the article and performed some of the operations. XW carried out conception, design, acquisition of data, and performed some of the operations. XZ carried out acquisition of data, analysis and interpretation of data and performed some of the operations. YZ carried out acquisition of data, critically revised the article and performed some of the operations. CL carried out acquisition of data, analysis and interpretation of data and participated some of the operations. All authors reviewed the final version of the manuscript and approved it for submission.

## Pre-publication history

The pre-publication history for this paper can be accessed here:

http://www.biomedcentral.com/1471-2377/11/52/prepub
